# Initial Operating Room Experience with Digital Variance Angiography in Carbon Dioxide-Assisted Lower Limb Interventions: A Pilot Study

**DOI:** 10.1007/s00270-020-02530-5

**Published:** 2020-05-31

**Authors:** Marcell Gyánó, Csaba Csobay-Novák, Márton Berczeli, István Góg, János P. Kiss, Krisztián Szigeti, Szabolcs Osváth, Balázs Nemes

**Affiliations:** 1 Department of Interventional Radiology, Heart and Vascular Center, Semmelweis University, Budapest, Hungary; 2 Department of Vascular Surgery, Heart and Vascular Center, Semmelweis University, Budapest, Hungary; 3 Kinepict Health Ltd, Budapest, Hungary; 4 Department of Vascular Surgery, Hungarian Defence Forces Medical Centre, Budapest, Hungary; 5 Department of Biophysics and Radiation Biology, Semmelweis University, Budapest, Hungary

**Keywords:** Digital variance angiography (DVA), Carbon dioxide (CO_2_) angiography, Image quality, Impaired renal function, Contrast-induced nephropathy (CIN), Lower limb interventions, Dose management, Iodine-free angiography

## Abstract

**Purpose:**

In retrospective clinical studies digital variance angiography (DVA) provided higher contrast-to-noise ratio and better image quality than digital subtraction angiography (DSA). Our aim was to verify the clinical usefulness and benefits of DVA in carbon dioxide (CO_2_)-assisted lower limb interventions.

**Materials and Methods:**

A workstation running the DVA software was integrated into a Siemens Artis Zee with Pure angiography system, and this new image processing technology was used in four patients (3 male, 1 female, age: 76.2 ± 4.2 years) with peripheral artery disease (PAD, Rutherford 2–3) and impaired renal function (average eGFR 25.5 ± 11.2 ml/min/1.73 m^2^). The DSA and DVA images of 46 CO_2_-assisted runs were visually evaluated by five experts in single-image evaluation using a 5-grade Likert scale and in paired comparisons.

**Results:**

DVA images received significantly higher score (3.84 ± 0.10) than DSA images (3.31 ± 0.10, *p* < 0.001). Raters preferred DVA images in terms of diagnostic value and usefulness for therapeutic decisions in 85.2% and 83.9% of all comparisons, respectively. These benefits were achieved at lower frame rates (1–3 FPS) than usually recommended for CO_2_ angiography (4–6 FPS). No adverse events were recorded during or after the procedures.

**Conclusions:**

Our initial experience shows that DVA might facilitate the correct diagnostic and therapeutic decisions, and potentially help to reduce radiation exposure in lower limb CO_2_ angiography. Although the dose management capabilities of DVA have to be validated in further clinical studies, this technology might be a useful new tool in the operating room and contributes to the safety and efficacy of CO_2_-enhanced endovascular interventions.

**Level of Evidence:**

Level IV.

**Electronic supplementary material:**

The online version of this article (10.1007/s00270-020-02530-5) contains supplementary material, which is available to authorized users.

## Introduction

Carbon dioxide (CO_2_) gas was introduced in medical imaging as a negative contrast agent decades ago [1]. Unlike iodinated contrast media (ICM), CO_2_ is a non-nephrotoxic and non-allergic contrast material, therefore the most important indications for its use are impaired renal function and ICM allergy [2]. According to a recent large-scale clinical study (AMACING), the occurrence of contrast-induced nephropathy (CIN) increases to fivefold in patients with eGFR < 30 ml/min/1.73 m^2^ [3], indicating a very high ICM-related risk for this subset of patients. The use of CO_2_ significantly decreased (− 65%) the incidence of CIN [4], and proved to be safe and effective in a 5-year follow-up study [5], thus CO_2_ might be an effective tool to prevent ICM-related side effects.

However, as CO_2_ is a negative contrast agent, obtaining good quality images with CO_2_ is a more challenging task, which requires a high-resolution DSA system with image stacking and CO_2_ specific acquisition modes to increase photon flux and enhance negative contrast visualization [2]. The stacking process requires higher frame rates (4–7.5 FPS), but even under these conditions, the additional use of ICM might be necessary sometimes, resulting in increased radiation exposure and risk for ICM-related adverse events. In special cases, the modified delivery of CO_2_ can improve the image quality [6], but a more general solution might be an improved image processing method.

The newly developed digital variance angiography (DVA) is based on the principles of kinetic imaging [7] and provides better image quality than the traditionally used digital subtraction angiography (DSA) [8]. In a recent multicentre study assessing the performance of DVA in lower limb CO_2_ angiography, the contrast-to-noise ratio (CNR) of DVA was 3.5–4.5 times higher than that of DSA, and the visual evaluations clearly showed the superiority of DVA [9]. This retrospective image processing study provided strong proof-of-concept validation but was not able to verify the usefulness and benefits of DVA in clinical practice. For this latter purpose, we have installed the image processing software in our operating room to investigate how the use of DVA can help CO_2_-enhanced lower limb interventions.

## Materials and Methods

### Patients

We present a case series of 4 patients who underwent elective CO_2_-enhanced lower limb angiography between May and July 2019 at the Heart and Vascular Center of Semmelweis University, Budapest, Hungary. The inclusion criteria were symptomatic peripheral artery disease (PAD, Rutherford 2–3) with severe renal impairment (eGFR < 30 ml/min/1.73 m^2^) or with moderate renal impairment (eGFR < 60 ml/min/1.73 m^2^) and earlier CIN. Every patient signed a written informed consent before the diagnostic or interventional procedure.

### Angiography Procedures

Transradial or transbrachial access and a 5F Pigtail-catheter at the level of aortic bifurcation was used for conventional angiography, whereas an antegrade femoral access was applied for femoro-popliteal interventions. An Angiodroid automated injector (Angiodroid SRL, Bologna, Italy) was used for CO_2_ delivery. In some cases, lower volumes (3–30 ml) of ICM (Ultravist 370, Bayer Schering Pharma, Berlin, Germany) were also used for diagnostic purposes. Images were recorded with a modified CO_2_ protocol (Siemens Evenflow, 1–3 FPS instead of the official preset 7.5). The use of the DVA method did not require additional radiation or contrast media load.

### Imaging System

**Fig. 1:**
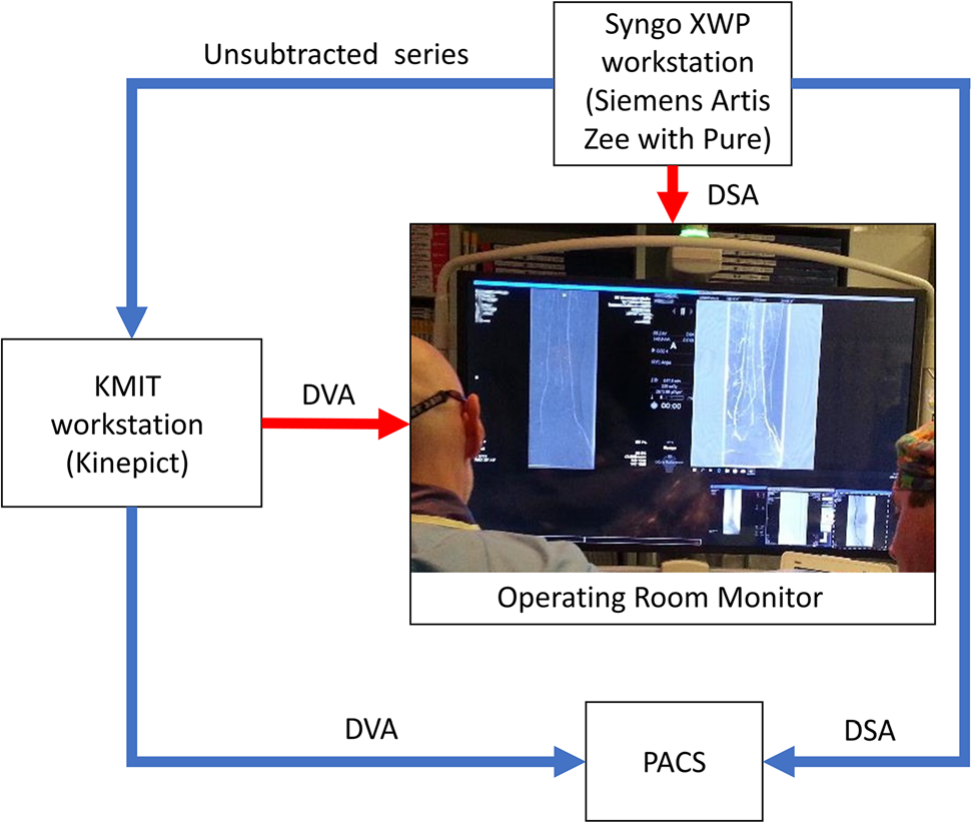
Integration of the Kinepict Medical Imaging Tool (KMIT) into the angiography suite. KMIT runs on an appropriately configured desktop (Intel Core i7 processor, 16 MB DDR4 RAM, 256 GB SSD). The Kinepict workstation receives the unsubtracted raw image series in DICOM format via the local area network (LAN) and sends back the processed files automatically to the operating room (OR) monitor via a video cable. The DVA image (right side of the monitor) appears within 1-2 s and can be used simultaneously with the DSA image (left side of the monitor). The presented DVA image can be postprocessed (pixel shift, brightness/contrast settings) on the OR screen, if necessary. For archiving purposes, the DVA files can be saved to the PACS via the LAN.* DVA* digital variance angiography,* DSA* digital subtraction angiography

DVA technology is available in a CE marked, platform-independent stand-alone software, the Kinepict Medical Imaging Tool v3.1 (KMIT, Kinepict Health Ltd, Budapest, Hungary), which was installed on a desktop computer. This workstation was integrated into a Siemens Artis Zee with Pure angiography suite containing a 30 × 40 cm detector and a Syngo workstation (XWP VD11B Service Pack 2, Siemens Healthcare, Munich, Germany). The KMIT workstation received the unsubtracted image series from the Syngo workstation via a DICOM port and the local area network, and sent back the DVA images automatically to the operating room monitor within 1–2 s, where the interventional radiologist could observe the DSA and DVA images simultaneously (Fig. 1), and used them for guiding the intervention.

### Visual Evaluation

The DSA and DVA images were saved for a retrospective visual evaluation. DSA images were carefully postprocessed (stacking, brightness/contrast optimization) to obtain the best available DSA quality and were compared with the DVA images generated automatically by the KMIT software. Five evaluators with 5+ years interventional radiology or endovascular surgery experience rated the image quality of all individual images in a blinded and randomized manner using a 5-grade Likert scale:

1:poor-5:outstanding (see Supplementary material for details). The mean ± SEM and, because of the non-Gaussian distribution of data, the median with interquartile ranges were calculated. Data were analyzed by the Wilcoxon signed rank test. The evaluators also compared the DSA-DVA image pairs in another blinded and randomized survey, where they had to select which image is more suitable for A: the identification of vascular pathology (diagnostic value); and B: achieving correct therapeutic decisions. To describe agreement between raters, percent agreement, Fleiss’ kappa and p values were calculated.

## Results

### Case Presentations

**Fig. 2:**
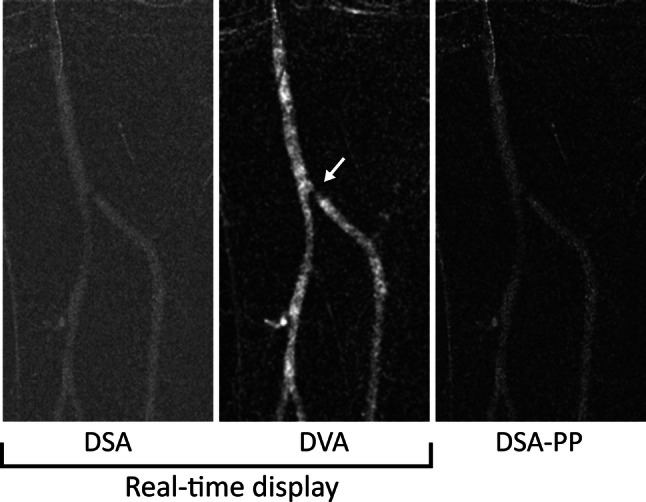
Case 1: Diagnostic lower limb angiography of a 77-year-old man with bilateral short distance claudication (Rutherford 3). The figure shows the magnified, left-sided tibial region. DVA gives a more detailed view of the high-grade stenosis (white arrow) at the origin of the anterior tibial artery. The DSA and DVA images (Evenflow preset at 1 FPS) appear automatically in the operating room monitor (real-time display), whereas the PP-DSA is visible only after manual postprocessing.* DVA* digital variance angiography,* DSA* digital subtraction angiography,* PP-DSA* postprocessed DSA, the best achievable DSA image after stacking and brightness/contrast adjustments

**Fig. 3:**
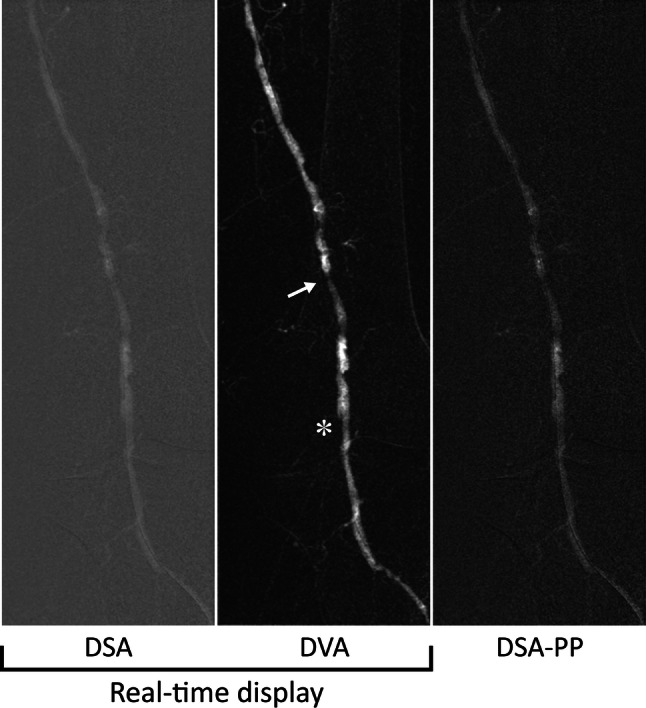
Case 2: Lower limb intervention of a 78-year-old man with left-sided short distance claudication (Rutherford 2). The figure shows the post-angioplasty results of an 8 cm long popliteal occlusion. DVA clearly visualizes a high-grade residual stenosis (white arrow) and a suspected flow-limiting dissection with visualized intima flap (white asterisk), whereas this pathology is not visible on the DSA images. The DSA and DVA images (Evenflow preset at 2 FPS) appear automatically in the operating room monitor (real-time display), whereas the PP-DSA is visible only after manual postprocessing.* DVA* digital variance angiography,* DSA* digital subtraction angiography,* PP-DSA* postprocessed DSA, the best obtainable DSA image after stacking and brightness/contrast adjustments

**Fig. 4:**
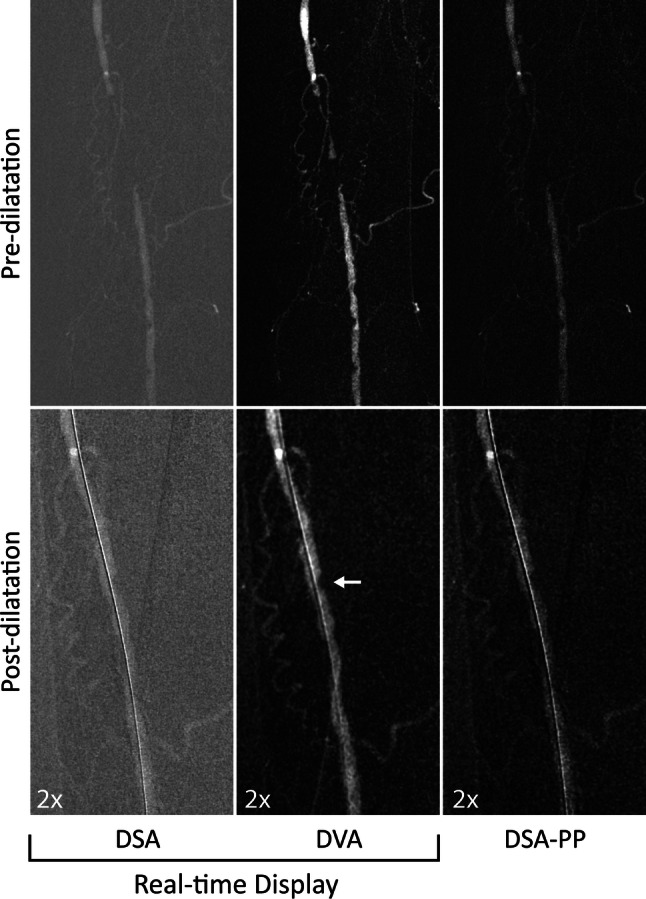
Case 3: Lower limb intervention of an 83-year-old woman with left-sided short distance claudication (Rutherford 3). Upper row: Pre-dilatation CO_2_ angiogram. The DVA image provides a more detailed view of the high-degree femoro-popliteal stenosis and the occlusion. The collaterals are also more visible. Lower row: Magnified post-dilatation image. DVA clearly shows the non-significant residual stenosis (white arrow). The DSA and DVA images (Evenflow preset at 1 FPS) appear automatically in the operating room monitor (real-time display), whereas the PP-DSA is visible only after manual postprocessing. DVA: digital variance angiography,* DSA* digital subtraction angiography,* PP-DSA* postprocessed DSA, the best obtainable DSA image after stacking and brightness/contrast adjustments

**Fig. 5:**
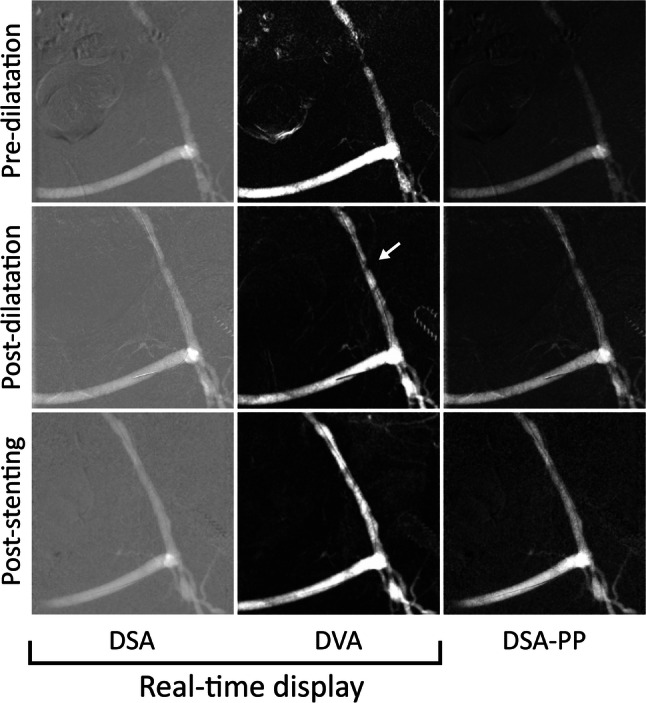
Case 4: Lower limb intervention of a 67-year-old man with short distance claudication (Rutherford 3). Upper row: High-grade stenosis on the left external iliac artery. A previous left-to-right femoro-femoral crossover bypass is also visible. Middle row: After the first ballooning, there was a significant residual stenosis (white arrow) that could be analyzed on the DVA image easier than on the DSA image. Lower row: Based on the DVA image, a stent was implanted with good result. The DSA and DVA images (Evenflow preset at 3 FPS) appear automatically in the operating room monitor (real-time display), whereas the PP-DSA is visible only after manual postprocessing.* DVA* digital variance angiography,* DSA* digital subtraction angiography,* PP-DSA* postprocessed DSA, the best achievable DSA image after stacking and brightness/contrast adjustments

All presented PAD patients (3 males, 1 female, age: 76.2 ± 4.2, creatinine (Cr) 260 ± 93 μmol/l, eGFR 25.5 ± 11.2 ml/min/1.73 m^2^) were referred by a vascular surgeon. None of the patients received hemodialysis care, and no adverse events were recorded during or after the procedures. The cases include one diagnostic lower limb angiography and three interventions (for details and images see Figs. 2, 3, 4, 5 and the Supplementary material). There was a general agreement among the interventional radiologists performing the procedures, that DVA images showed more details and better helped the interventional decisions. To objectivate this opinion, the image set of the 4 patients (altogether 46 DSA-DVA image pairs) were retrospectively evaluated by 5 experienced experts.

### Visual Evaluation

**Fig. 6:**
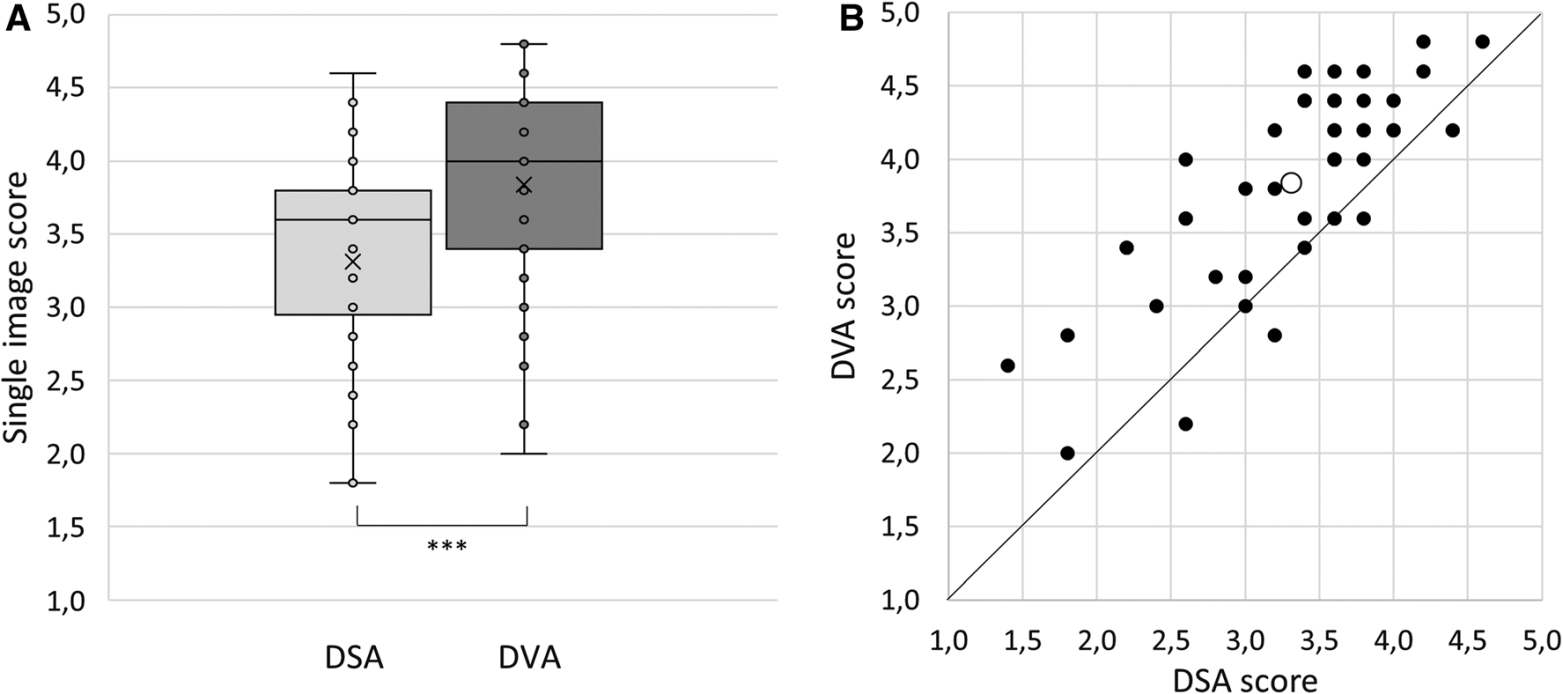
Comparison of single-image evaluation scores. A: The box and whisker plot shows the median (line), the mean (*x*) and the interquartile range (box) of each group. Data were analyzed by the Wilcoxon signed rank test (***p < 0.001). B: DVA-DSA image score plot. Each point represents an individual image pair. The abscissa and the ordinate represent the DSA and DVA scores, respectively. The open circle shows the mean DSA-DVA score of the 46 image pairs. Abbreviations: DSA: digital subtraction angiography; DVA: digital variance angiography

In *single-image evaluation*, DVA images received significantly higher score (mean ± SEM: 3.84 ± 0.10) than DSA images (3.31 ± 0.10, Wilkinson signed rank *p* < 0.001). The data did not follow a normal distribution, therefore the median and interquartile range were also determined for DVA (4.00, 3.45-4.40) and DSA (3.6, 3.00-3.80) (Fig. 6A). To compare the DSA and DVA scores of individual images, a DVA-DSA plot was prepared (Fig. 6B). In 84.8% of images DVA received higher score, in 6.5% the score was equal, and DSA received higher score only in 8.7%.

In *paired comparisons,* the evaluators preferred DVA images in terms of diagnostic value and usefulness for therapeutic decisions in 85.2% and 83.9% of all comparisons, respectively. The percent agreement, Fleiss K and p were 79.6%, 0.21, *p* < 0.001 and 77.8%, 0,18, *p* < 0.001, respectively.

## Discussion

DSA records a contrast-enhanced image series and subtracts one of these images (the mask) from the other frames. These subtracted images compose the DSA video and their appropriate integration yields the DSA image [10]. In contrast, DVA uses all frames of a series and calculates standard deviation for each pixel [7]. This algorithm enhances the functional motion-related information (i.e., the flow of contrast agents) but suppresses the noise, therefore the CNR and consequently the image quality is greatly improved.

In line with this, the image comparison showed that the identification of vascular pathology and the therapeutic decisions were much easier on the basis of DVA images. The real-time DVA images received significantly higher Likert score than the postprocessed DSA images, and the available postprocessing tools (brightness/contrast adjustment, pixel shift) make the technology even more versatile. It should be emphasized that the image quality was improved at a lower frame rate (1–3 FPS) than the factory preset 7.5 FPS or the generally recommended 4–6 FPS [2], which provides opportunity for substantial radiation exposure reduction in CO_2_ angiography.

## Conclusions

Our initial experience shows that DVA might facilitate the correct diagnostic and therapeutic decisions and potentially help to reduce radiation exposure in lower limb CO_2_ angiography. Although the dose management capabilities of DVA have to be validated in further clinical studies, our preliminary results clearly demonstrate that the platform-independent KMIT software might be a useful new tool in the operating room, and contributes to the safety and efficacy of CO_2_-enhanced endovascular interventions.

## Electronic supplementary material

Below is the link to the electronic supplementary material.

Supplementary material 1 (DOCX 17 kb)
