# Supplementary material for: Initial Operating Room Experience with Digital Variance Angiography in Carbon Dioxide-Assisted Lower Limb Interventions: A Pilot Study
Source: Cardiovasc Intervent Radiol. 2020 May 31;43(8):1226–31. doi: 10.1007/s00270-020-02530-5 (PMC7369257; doi:10.1007/s00270-020-02530-5)
Supplement: Supplementary file 1 — Supplementary material 1 (DOCX 17 kb) [file 270_2020_2530_MOESM1_ESM.docx]

**Supplemental material/1**

**Description of the Likert scale used in the visual evaluation:**

Five evaluators with 5+ years interventional radiology or endovascular surgery experience rated the image quality of all individual images in a blinded and randomized manner using a 5-grade Likert scale:

1: Poor image quality, vascular structures are not distinguishable

2: Low image quality, vascular structures are distinguishable but not suitable for diagnosis

3: Medium image quality, large vessels are well visible, but smaller arteries and stenotic lesions cannot be reliably evaluated

4: Good image quality, both large and small arteries are well visible, suitable for diagnostic and interventional decisions (daily routine level)

5: Outstanding image quality, provides more details and easier diagnostic decisions than the daily routine images

The mean ± SEM and, because of the non-Gaussian distribution of data, the median with interquartile ranges were calculated.

**Supplemental material/2**

**Medical and technical details**

**Case 1:** Diagnostic lower limb angiography of a 77-year-old man with bilateral short distance claudication (Rutherford 3) and a history of hypertension and chronic renal failure (Cr:325 μmol/l, eGFR:16 ml/min/1.73m2). Following the MR angiography, a non-selective X-ray angiography (from transradial access) was necessary because of motion artefacts, and a bilateral femoro-popliteal stenosis was diagnosed without ICM usage. Figure 2 (see article) shows the magnified, left-sided tibial region. DVA gives a more detailed view of the high-grade stenosis (white arrow) at the origin of the anterior tibial artery (Evenflow preset at 1 FPS). Access from the left radial artery, 5F pigtail catheter (125 cm), first shot at the level of renal arteries, second shot just above the bifurcation, further injection from this non-selective position. Injection parameters: 500 Hgmm, 60 ml CO2, total CO2 usage 375 ml. Evenflow preset at 1 FPS

**Case 2:** Lower limb intervention of a 78-year-old man with left-sided short distance claudication (Rutherford 2) and severe renal impairment (Cr:395 μmol/l, eGFR:13 ml/min/1.73m^2^). An 8 cm long popliteal occlusion was recanalized via the subintimal technique from a femoral antegrade access. Figure 3 (see article) shows the post-angioplasty results. DVA clearly visualizes a high-grade residual stenosis (white arrow) and a suspected flow-limiting dissection with visualized intima flap (white asterisk), whereas this pathology is not visible on the DSA images. Based on the DVA image we decided to implant a stent. During the procedure 3 ml ICM (Ultravist 370) was used (Evenflow preset at 2 FPS). Anterograd femoral access, 5F short sheat (13 cm), Balloon dilatation (6 mm x 60 cm), then self-expandable stent (8 mm x 40 cm). Injection parameters: at the middle segment of the superficial femoral artery, 350 Hgmm 40 ml CO2, total CO2 usage 200 ml. Additional ICM (a single 3 ml injection) was used to observe the potential dissection.

**Case 3:** Lower limb intervention of a 78-year-old man with left-sided short distance claudication (Rutherford 2) and severe renal impairment (Cr:395 μmol/l, eGFR:13 ml/min/1.73m^2^). An 8 cm long popliteal occlusion was recanalized via the subintimal technique from a femoral antegrade access. Figure 4 (see article) shows the post-angioplasty results. DVA clearly visualizes a high-grade residual stenosis (white arrow) and a suspected flow-limiting dissection with visualized intima flap (white asterisk), whereas this pathology is not visible on the DSA images. Based on the DVA image we decided to implant a stent. During the procedure 3 ml ICM (Ultravist 370) was used (Evenflow preset at 2 FPS). Antegrad femoral access, 5F short sheat (13 cm), Balloon dilatation (6 mm x 60 cm). Injection parameters: at the middle segment of the superficial femoral artery, 350 Hgmm 40 ml CO2, total CO2 usage 495 ml, additional ICM (30 ml) was used to observe the residual stenoses. This patient had only mild renal impairment (eGFR 48 ml/min/1.73m^2^).

**Case 4:** Lower limb intervention of a 67-year-old man with short distance claudication (Rutherford 3) and impaired renal function (Cr:226 μmol/l, eGFR:25 ml/min/1.73m^2^). The medical history contained an uncharacterized autoimmune deficiency with joint inflammations and vascular lesions, hypertension, five coronary interventions and left-to-right femoro-femoral crossover bypass. The left external iliac artery stenosis was recanalized from transbrachial access. Figure 5 (see article) shows a high-grade stenosis on the left external iliac artery. The femoro-femoral crossover bypass is also visible (upper row). After the first ballooning, there was a significant residual stenosis (white arrow) that could be analyzed on the DVA image easier than on the DSA image (middle row). Based on the DVA image, a stent was implanted with good result (lower row). During the intervention no ICM was used (Evenflow preset at 3 FPS). Access from the right brachial artery, 4F pigtail catheter (100 cm). Diagnostic shots (see Case 1). After the diagnostic phase, switch to 6F sheat (90 cm). Balloon dilatation (6 mm x 60 cm), then self-expandable stent (8 mm x 40 cm). Injection parameters: following the diagnostic phase just above the lesion, 500 Hgmm, 60 ml CO2, total CO2 usage 375 ml.
